# Physiological and proteomic analyses on artificially aged *Brassica napus* seed

**DOI:** 10.3389/fpls.2015.00112

**Published:** 2015-02-25

**Authors:** Xiaojian Yin, Dongli He, Ravi Gupta, Pingfang Yang

**Affiliations:** ^1^Key Laboratory of Plant Germplasm Enhancement and Specialty Agriculture, Wuhan Botanical Garden, Chinese Academy of SciencesWuhan, China; ^2^Department of Plant Bioscience, College of Natural Resources and Life Science, Pusan National UniversityMiryang, South Korea

**Keywords:** *Brassica napus*, seed aging, controlled deterioration treatments, proteomics

## Abstract

Plant seeds lose their viability when they are exposed to long term storage or controlled deterioration treatments, by a process known as seed aging. Based on previous studies, artificially aging treatments have been developed to accelerate the process of seed aging in order to understand its underlying mechanisms. In this study, we used *Brassica napus* seeds to investigate the mechanisms of aging initiation. *B. napus* seeds were exposed to artificially aging treatment (40°C and 90% relative humidity) and their physio-biochemical characteristics were analyzed. Although the treatment delayed germination, it did not increase the concentration of cellular reactive oxygen species (ROS). Comparative proteomic analysis was conducted among the control and treated seeds at different stages of germination. The proteins responded to the treatment were mainly involved in metabolism, protein modification and destination, stress response, development, and miscellaneous enzymes. Except for peroxiredoxin, no changes were observed in the accumulation of other antioxidant enzymes in the artificially aged seeds. Increased content of abscisic acid (ABA) was observed in the artificially treated seeds which might be involved in the inhibition of germination. Taken together, our results highlight the involvement of ABA in the initiation of seed aging in addition to the ROS which was previously reported to mediate the seed aging process.

## Introduction

Successful germination of seeds is a prerequisite for plants to initiate their life cycle and to distribute their progeny, and is largely determined by the seed vigor (Holdsworth et al., 2008; Rajjou et al., 2012). Seed aging is a process which results in delayed germination, reduction in germination rate, and sometimes even a total loss of seed viability (Priestley, 1986). Prolonged storage of seeds induces seed aging which is a major complication in plant germplasm conservation (Garza-Caligaris et al., 2012). In agriculture, the aged crop seeds germinate poorly and negatively affect the seedlings growth and eventually the yield (Ellis, 1992). When stored in uncontrolled conditions, most of the agricultural crops have 1–5 years of seed viability, which is much less when compared to the wild plants. Optimized storage conditions have been proved fruitful in slowing down the rate of seed aging and eventually increasing the seed life span. For orthodox seeds, low temperature and moisture content are helpful (Walters et al., 2005) while high temperature and humidity have been shown to induce and accelerate the seed aging process (El-Maarouf-Bouteau et al., 2011).

Seed aging leads to various cellular and metabolic alterations including loss of membrane integrity, degradation of DNA, reduced primary metabolism and so on (Corbineau et al., 2002; Kibinza et al., 2006; El-Maarouf-Bouteau et al., 2011). Although the mature seeds are physiological quiescent, these could not prevent the production of reactive oxygen species (ROS). The over-accumulation of ROS and its attack on lipids and proteins are supposed to be the major cause of seed aging (Bailly, 2004). ROS result in the peroxidation and degradation of lipids, which eventually damage the integrity of cellular membranes (Lee et al., 2012; Parkhey et al., 2012). Generally, ROS are regarded as the main factor that leads to seed aging during storage (Priestley, 1986). It has been shown that accumulation of hydrogen peroxide is related to the loss of seed viability in sunflower (Bailly et al., 1996; Kibinza et al., 2006). Under stress conditions, ROS promote program cell death (PCD) in both plants and animals (Grant and Loake, 2000; Neill et al., 2002). However, it is still unknown whether the seed aging is also induced by the ROS through the triggering of PCD or not.

Seed aging is highly associated with the storage conditions, however, recent studies have shown that seeds of different plant species show varied rate of seed aging under the same storage conditions (Walters et al., 2005). It is believed that the viability of the seed is determined by its genetic background as well as the storage conditions (Bewley, 1997; Miura et al., 2002; Clerkx et al., 2004). In Arabidopsis, genes involved in flavonoid and tocopherol biosynthesis can contribute to its seed longevity (Debeaujon et al., 2000; Sattler et al., 2004). Furthermore, a dormancy-related gene *delayed of germination 1* (*DOG1*) (Bentsink et al., 2006) and a heat stress responsive transcription factor (Prieto-Dapena et al., 2006) were also found to improve the resistance to aging. Very recent studies verified that methionine sulfoxide reductases from *Medicago truncatula* and PROTEIN L-ISOASPARTYL METHYLTRANSFERASE (PIMT) from Arabidopsis and lotus (*Nelumbo nucifera*) could enhance the seed vigor and longevity (Oge et al., 2008; Chatelain et al., 2013; Verma et al., 2013). With the advancement in genomics and other large scale “omics” techniques, number of transcriptomics and proteomics studies have been conducted in the last decade in order to identify and characterize the potential biomarkers for the seed aging (Nakabayashi et al., 2005; Rajjou et al., 2008).

*Brassica napus* is one of the major sources of edible oil all over the world. However, *B. napus* seeds are harvested in late spring, and their storage go through the summer season, which leads to the loss of their viability. Thus, it is of great practical importance to prevent the loss of seed vigor, which needs to obtain a comprehensive understanding of the mechanisms underlying the seed aging. Unfortunately, study on this topic in *B. napus* is rather weak. In this study, we exposed the *B. napus* seeds to high temperature and humidity, and conducted a comparative proteomic analysis of control and artificial aged seeds in order to understand the underlying mechanisms. A lot of differentially accumulated proteins were identified, which were different with previous studies in other plants. Our results provide some new insights on seed aging mechanisms in *B. napus*.

## Materials and methods

### Plant growth, aging treatments and germination assays

*B. napus* (zhongshuang11) plants were grown in green house under natural light condition in Wuhan, China. The non-dormant seeds were harvested in May of each year and used as experimental materials. Freshly harvested *B. napus* seeds were treated with high temperature and humidity according to Rajjou et al. (2008) with slight modifications. Briefly, seeds were exposed to 40°C and 90% air humidity for different time points (0, 12, 24, and 48 h). Seeds stored at room temperature in sealed plastic bag, at dry conditions, for 1 year were used as natural aged seeds.

The untreated and treated seeds were dried in oven at 40°C overnight, and then dipped in distilled water at 26°C in darkness for germination. The germination rate for each sample was calculated after every 6 h until there is no more seed germination. For abscisic acid (ABA) treatment and gibberellic acid (GA) recovery experiments, seeds were imbibed with 10^−8^ M ABA and 10^−7^ M GA_3_ solutions during germination, respectively. Three biological replicates were performed for each treatment as well as germination assay with 50 seeds in each set of the replicate. The schematic flowchart of the whole experiment is shown in Figure S1.

### Measurement of ion leakage, malondialdehyde and hydrogen peroxide content

The ion leakage was calculated as described previously (Shi et al., 2012), by measuring the relative conductivity of the samples. Briefly, 0.1 g of seeds at 0 h of germination for both samples were incubated in 6 mL of distilled water, for 4 h at room temperature with constant shaking. After the incubation, the initial conductivity (C1) of the solution was measured. Final conductivity (C2) of the solution was measured after boiling the seeds for 30 min and cooling down the solution to room temperature. REL was calculated as the percentage of conductivity before and after boiling [(C1/C2) × 100] using a conductivity meter (Leici-DDS-307A, Shanghai precision scientific instrument company, Shanghai, China).

The malondialdehyde (MDA) content was measured using a commercial kit (S0131, Beyotime, Nanjing, China) according to the manufacturer's protocol, which is based on the reaction between MDA and thiobarbituric acid to produce a red compound. In brief, 0.2 g of seeds were homogenized with 2 mL of ice-cold phosphate buffer and centrifuged at 1600 × *g* for 10 min at 4°C. The supernatant was then mixed with an equal volume of 0.5% thiobarbituric acid solution. The mixture was boiled for 10 min. After being cooled down to room temperature with water, the mixture was centrifuged at 3000 × *g* for 15 min at room temperature. The absorbance of the supernatant was determined at 530 nm. The concentration of MDA was calculated according to standard curve which was generated with known concentrations of MDA.

Measurement of H_2_O_2_ was carried out according to the method described before (Jaw-Neng Lin, 1998). In brief, 0.1 g of seeds were homogenized in PBS buffer and centrifuged at 12000 × *g* for 20 min at 4°C. The supernatant was mixed with equal volume of 0.1% titanium sulfate in 20% H_2_SO_4_ (v/v), and then centrifuged again at 6000 × *g* for 15 min at room temperature. The absorbance of the supernatant was measured at 410 nm. The concentration of H_2_O_2_ was calculated based on the standard curve which was made with a series of H_2_O_2_ solutions with known concentration. All the measurements were conducted for three biological replicates.

### Measurement of abscisic acid content

ABA concentration was measured using a derivatization approach coupled with nano-LC-ESI-Q-TOF-MS (Bruker Daltonics, Bremen, Germany) as described previously (Chen et al., 2012). Briefly, 0.1 mg of seeds were homogenized in liquid nitrogen, and then transferring the powder to a 2 mL centrifuge tube, followed by extraction with 500 μL modified Bieleski solvent (methanol/water/formic acid, 15/4/1, v/v/v) was added to it and the mixture was incubated at 4°C for 12 h. The stable isotope labeled ABA ([^2^H_6_] ABA, 50 ng/g) was added to each of the samples to serve as internal standards for the quantification. Then, the supernatants were sequentially passed through the tandem solid phase extraction (SPE) cartridges containing C18 adsorbent (50 mg) and SAX adsorbent (200 mg). Before SPE extraction, the tandem cartridges were pre-conditioned with 8 mL H_2_O, 8 mL methanol, and 8 mL modified Bieleski solvent. After sample loading, the C18 cartridge was removed and the SAX cartridge was rinsed with 2 mL methanol/H_2_O (20/80, v/v). After that, 3 mL acetonitrile (CAN) with 1% Hydrofluoric acid (FA) (v/v) was applied to elute the targeted ABA and the eluent was evaporated under mild nitrogen stream at 35°C followed by re-dissolving in 100 μL H_2_O. The resulting solution (100 μL) was then acidified with 10 μL FA, and extracted with ether (2 × 1 mL). The ether phase was combined, dried under nitrogen gas and reconstituted in 100 μL ACN. To the resulting solution, 10 μL triethylamine (TEA) (20 μmol/mL) and 10 μL 3- Bromoactonyltrimethylammonium bromide (BTA) (20 μmol/mL) were added. The reaction solution was vortexed for 30 min at 35°C and evaporated under nitrogen gas followed by re-dissolving in 200 μL H_2_O/ACN (90/10, v/v) for instrumental analysis. The calibration curve was constructed by comparing peak area ratio (analyte/IS) to concentrations. The content of ABA was calculated according to the calibration curve. Three biological replicates were conducted.

### Superoxide dismutase and catalase activity assays

Total superoxide dismutase (SOD) activity was measured using a commercial WST-1 kit (S0102, Beyotime) following manufacturer's protocol. Briefly, seeds were powdered using liquid nitrogen and then homogenized in phosphate balanced solution buffer (PBS, pH7.5). The mixture was centrifuged at 12,000 × *g* at 4°C for 15 min. The supernatant thus obtained was used for the SOD activity measurement. The principle of this method lied on the coupling of 2-(4-iodophenyl)-3-(4-nitrophenyl)-5-(2, 16 4-disulfophenyl)-2H-tetrazolium (WST-1) with xanthine oxidase (XO) to generate O^2−^ and formazan dye, which can be inhibited by SOD through catalyzing O^2−^ into H_2_O_2_ and O_2_. SOD activity can be calculated by determining the absorbance of formazan dye at 450 nm.

Catalase (CAT) activity was assayed using commercial kit (S0051, Beyotime) as described previously (Shi et al., 2012). Briefly, 10 μL of 250 mM H_2_O_2_ was mixed with 5 μL of protein supernatant. The H_2_O_2_ was decomposed by CAT for 5 min, and the remaining H_2_O_2_ coupled with a substrate was treated with peroxidase (POD) to generate N-4-antipyryl-3-chloro-5-sulfonate-p-benzoquinonemonoimine. CAT activity was determined by calculating the decomposition rate of H_2_O_2_at 520 nm. Both enzymes were assayed for three replicates.

### Protein extraction and two-dimensional electrophoresis

Proteins were extracted from *B. napus* seeds at 0 and 18 h after imbibition according to the method described previously (Chi et al., 2010). Briefly, 0.2 g of seeds were homogenized in ice-cold buffer containing 20 mM Tris-HCl (pH 7.5), 250 mM sucrose, 10 mM ethylenebis(oxyethylenenitrilo) tetraacetic acid (EGTA), 1 mM phenylmethanesulfonyl fluoride (PMSF), 1 mM DL-dithiothreitol (DTT), and 1% Triton X-100. The homogenate was centrifuged at 12000 × *g* for 30 min at 4°C. The supernatant was mixed with isometric Tris-Phenol (pH7.8) and vortexed for 20 min. Mixture was then centrifuged at 12000 × *g* for 15 min at 4°C. After centrifugation, the supernatant phenol phase and intermediate denatured protein layer were collected. Phenol phase containing proteins was then mixed with 5 volumes of 0.1 M ammonium acetate in methanol and incubated at −20°C overnight. Pellet, obtained after centrifugation at 12000 × *g* for 30 min, was washed 4 times with ice-cold acetone and vacuum dried. For two-dimensional electrophoresis (2-DE), the dried proteins pellets were dissolved in rehydration buffer containing 7 M urea, 2 M thiourea, 4% 3-[(3-Cholamidopropyl)dimethylammonio]propanesulfonate (CHAPS), 0.2% carrier ampholyte, and 65 mM DTT, and quantified according to Bradford's method (Bradford, 1976; Kruger, 1994). A total of 600 μg of proteins of each sample were loaded on 17 cm IPG strip by rehydration loading for 12 h at room temperature. Based on primary screening, the IPG strips with pH 5–8 (linear) were selected in this study. Isoelectric focusing (IEF) was carried out at 200, 500, and 8000 V for 1, 1.5, and 10.5 h, respectively (He et al., 2011). After IEF, the strips were incubated in equilibration buffer containing 0.05 M Tris-HCl pH 6.8, 2.5% SDS, 10% (v/v) glycerol and 2% DTT and shaken for 15 min, and then for another 15 min with the iodoacetamide replaced DTT equilibration buffer. The second-dimensional separation of the proteins was carried out on 12% SDS-PAGE (Li et al., 2012).

### Gel staining, scanning and analysis

The gels were stained with Coomassie brilliant blue-red (CBB-R) 250 for 40 min, and then destained with 20% ethanol containing 10% acetic acid. The destained gels were scanned using Epson Perfection™ V700 Photo scanner (Epson, China Co., Ltd.) at 800 dots per inch (dpi) resolution. The transparency mode was used to obtain a gray scale image. The images were digitized and analyzed with PDQuest™ 2-DE Analysis Software 8.0 (BIO-RAD, CA, USA). The relative volume of each spot, which is defined as is the ratio between the volume of the given spot and the total volume of all the spots displaying on the gel, was used to represent the corresponding protein abundance. To obtain reproducible result, three biological repeat experiments were conducted. All the 12 gels were digitized separately. Automatic matching between different gels was conducted with four spots as internal standards. After that, manual adjustment was carried out to avoid mismatching. Spots which were detected in all the three replicates were used for comparative analysis. The spots showing more than 2-fold changes in abundance were defined as differentially displayed protein spots.

### Identification of proteins through MALDI-TOF/TOF MS

Differentially displayed protein spots were excised from the gels and distained using 50 mM NH_4_HCO_3_ in 50% (v/v) ACN. After complete de-staining, excised spots were first dehydrated using 50 μL 100% ACN and then rehydrated with 10 pmol trypsin in 25 mM NH_4_HCO_3_ at 4°C for 1 h. Trypsin digestion was carried out at 37°C overnight. After digestion, the peptides were extracted according to the method described before (Yang et al., 2007). The collected peptides were desalted and analyzed with an ultrafleXtreme Matrix-Assisted Laser Desorption/ Ionization tandem Time of Flight (MALDI-TOF/TOF) mass spectrometer (Bruker, Germany) in a MS-MS mode. All the parameters were set to default. Briefly, 0.5 kHz laser with 40 k resolution was applied. The flexAnalysis software (Bruker) was used to generate the peak lists and process the MS and MS/MS spectra, which were searched against NCBInr (containing 15823071 sequences and 5433757279 residues) and Swiss-Prot databases (containing 138011 sequences from *B. napus*) using MASCOT as a search engine (Mascot Wizard 1.2.0, Matrix Science Ltd.) through BioTools (version 3.2) interface. The search parameters were set as follows: taxonomy, Viridiplantae; fixed modifications, carbamidomethylation; variable modification, methionine oxidation; MS tolerance, 50 ppm; MS/MS tolerance, 0.5 Da, peptide mass, monoisotopic. Only the significant hits (*p* < 0.01) with peptide scores >45 were accepted.

### Statistical analysis

The statistical analyses were carried out using Student's *t*-test when only two groups were compared, or with One-Way ANOVA followed by Tukey's multiple comparisons test for all other comparisons based on the three independent replicates.

## Results

### Determination of suitable artificial aging treatments

**Figure 1:**
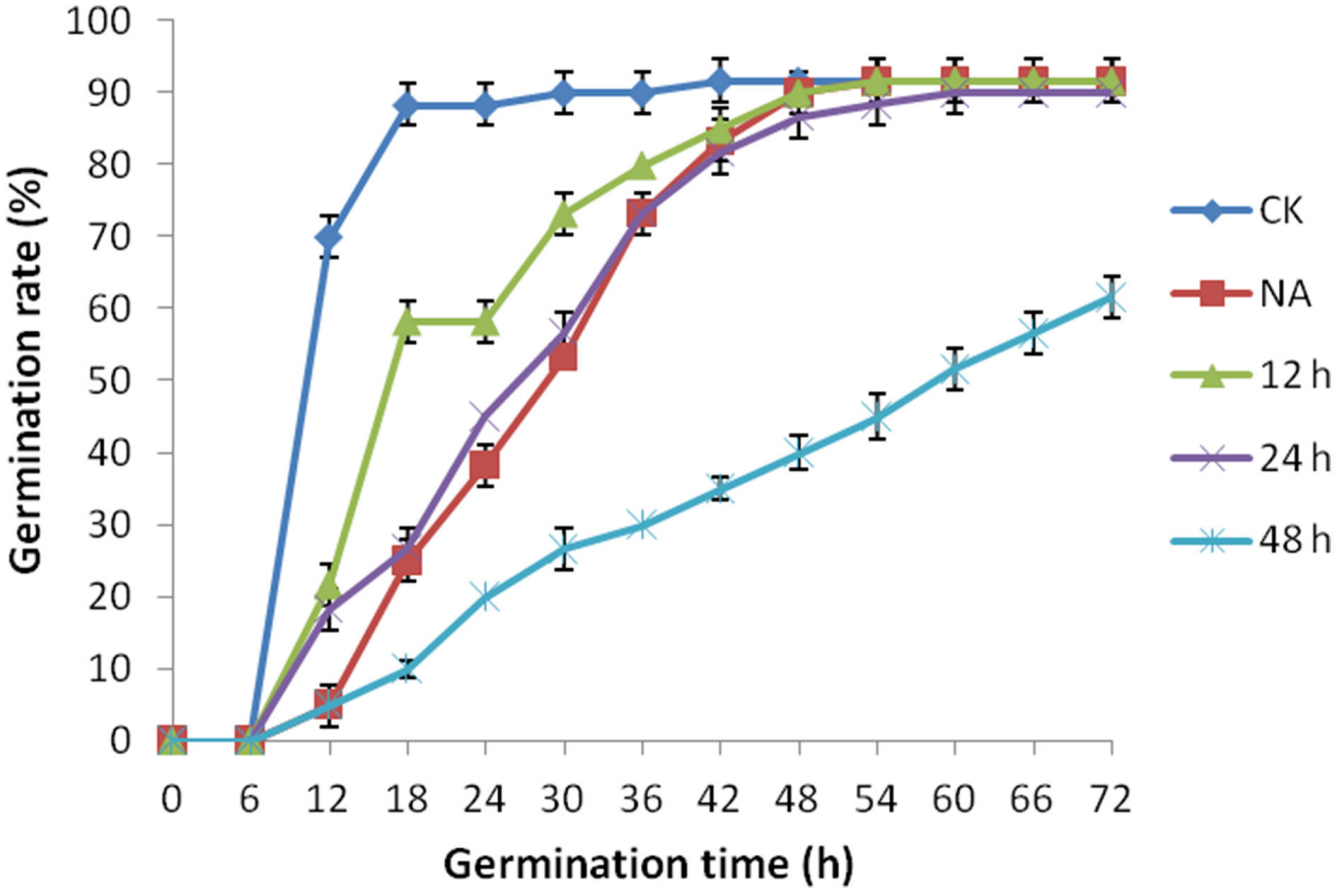
**The effect of artificial aging treatments on *Brassica napus* seed germination**. CK and NA stand for the control and natural aged seeds (stored for 1 year). And 12, 24, and 48 h stand for seeds subjected to artificially aging treatments with 40°C and 90% relative humidity for 12, 24, and 48 h respectively.

Mature seeds of *B. napus* gradually lose their viability during long term storage, which is defined as natural aging. To better understand the process of natural aging, germination percentages of 1 year aged seeds and freshly harvested seeds were calculated. Freshly harvested seeds started germination just after 6 h of imbibition and took less than 12 h for 50% percent of the seeds to germinate (Figure 1). In contrast, germination of the aged seeds was clearly delayed (Figure 1). It took about 30 h for the 1 year aged seeds to achieve 50% germination. In-spite of delayed germination of the aged seeds, most of the seeds were still able to germinate. Based on these results, we concluded that these seeds were at the early stage of natural aging and thus could be used as criterion to determine the suitable artificial aging treatments.

The fresh harvested seeds were exposed to 40°C and 90% relative humidity for 0, 12, 24, and 48 h and viability of each seed sample was checked using germination assays (Figure 1). The treatment apparently declined the seed germination rate with increasing exposure time (Figure 1). For the sake of comparison, sample with similar germination rate with the natural aged control was selected for further study. Based on this criterion, 24 h of treatment (Figure 1) was determined as suitable artificial aging treatment as its germination rate was almost similar to that of natural aged seeds. Hereafter, treatment with 40°C and 90% relative humidity for 0 and 24 h is defined as control (CK) and CDT treatment, respectively in the rest of the manuscript.

### Effects of artificial aging on physio-biochemical status of *B. napus* seed

**Figure 2:**
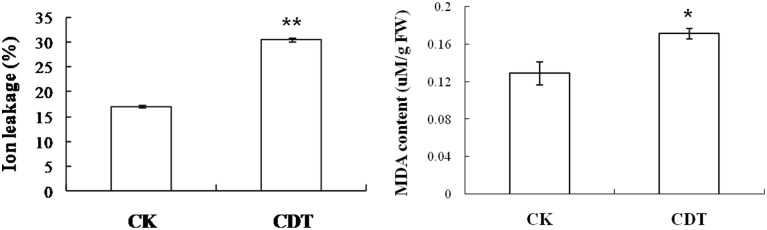
**The effect of CDT on the ion leakage and MDA content in the seeds at 0 h of germination**. Values are the means ± SE from three biological replicates. ^*^ and ^**^ indicate significant difference at *P* < 0.05 and 0.01 respectively.

**Figure 3:**
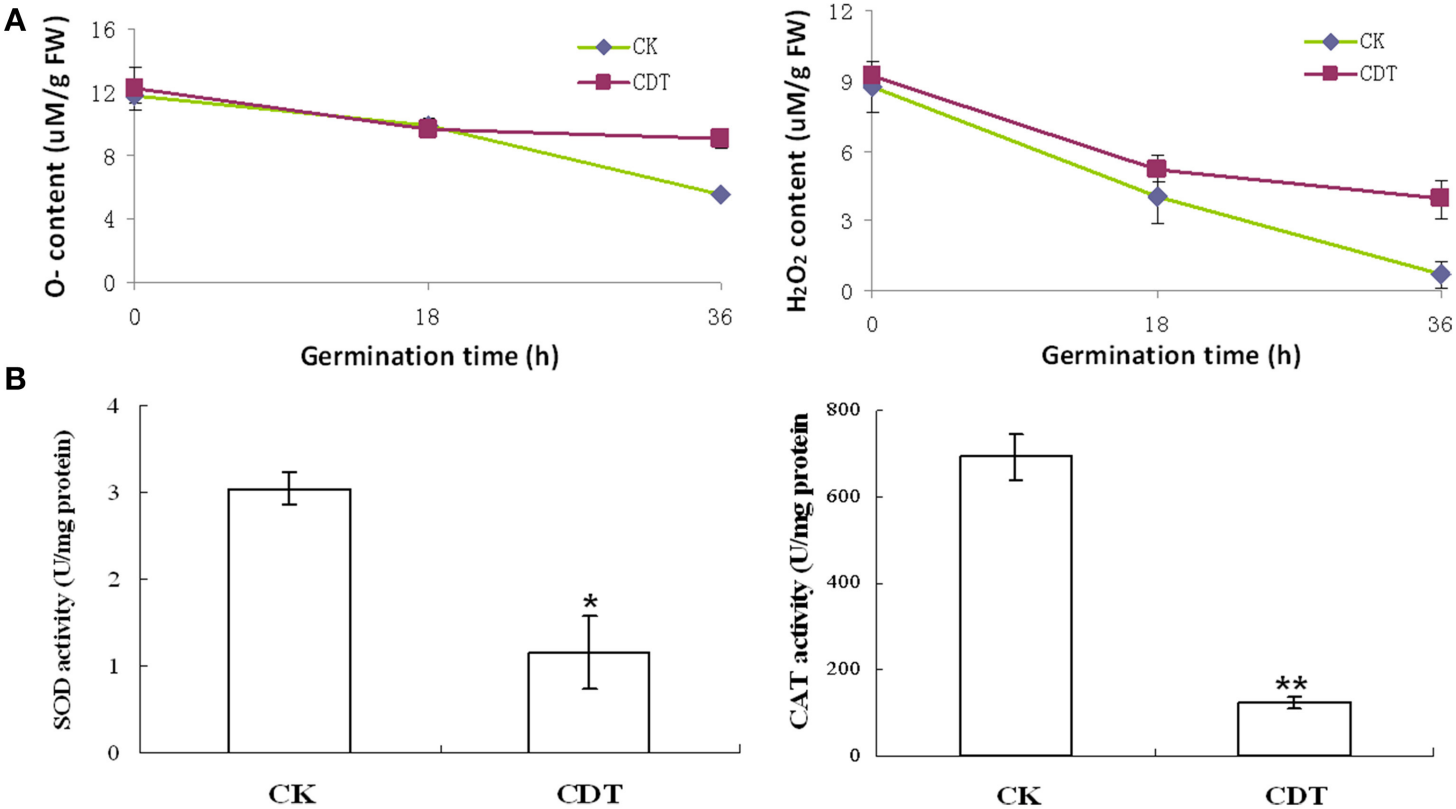
**The effects of CDT on the ROS homeostasis**. **(A)** ROS content, including O^−^ (left panel) and H_2_O_2_ (right panel). **(B)** Changes in the SOD (left panel) and CAT (right panel) activity during germination. Values are the means ± SE from three biological replicates. ^*^ and ^**^ indicate significant difference at *P* < 0.05 and 0.01 respectively.

As mentioned above, plasma membrane could be damaged during aging (Lee et al., 2012; Parkhey et al., 2012), therefore, in order to test the integrity of the membranes, relative ion leakage of CK and CDT samples at 0 h of imbibition were measured. The CDT samples showed approximately 1.8 fold higher ion leakage than the CK samples, indicating higher membrane damage in the former sample (Figure 2). Moreover, MDA content measurements of CK and CDT seeds also showed an increased MDA in the CDT seeds (Figure 2), further suggesting the increased membrane damage in the CDT seeds. Besides ion-leakage and MDA content measurements, we also measured the concentrations of H_2_O_2_ and O^−^, which are considered the two major ROS in plants. Surprisingly, the concentrations of both H_2_O_2_and O^−^ were found to be similar in CK and CDT samples, indicating that increased ROS concentration might not be a necessary event during seed aging of *B. napus* seeds (Figure 3A). A gradual decrease in the concentration of ROS was observed during seed germination (Figure 3A). However, the decrease of ROS in the artificial aged seeds was much slower as compared to the control sample (Figure 3A), which might explain why the germination in aged seeds was delayed. Consistent with the changes in the ROS concentrations, the activities of the antioxidant enzymes, SOD, and CAT, were also dramatically declined in the artificially aged seeds before germination (Figure 3B).

### Changes in the proteome of *B. napus* seeds exposed to artificial aging treatment

**Figure 4:**
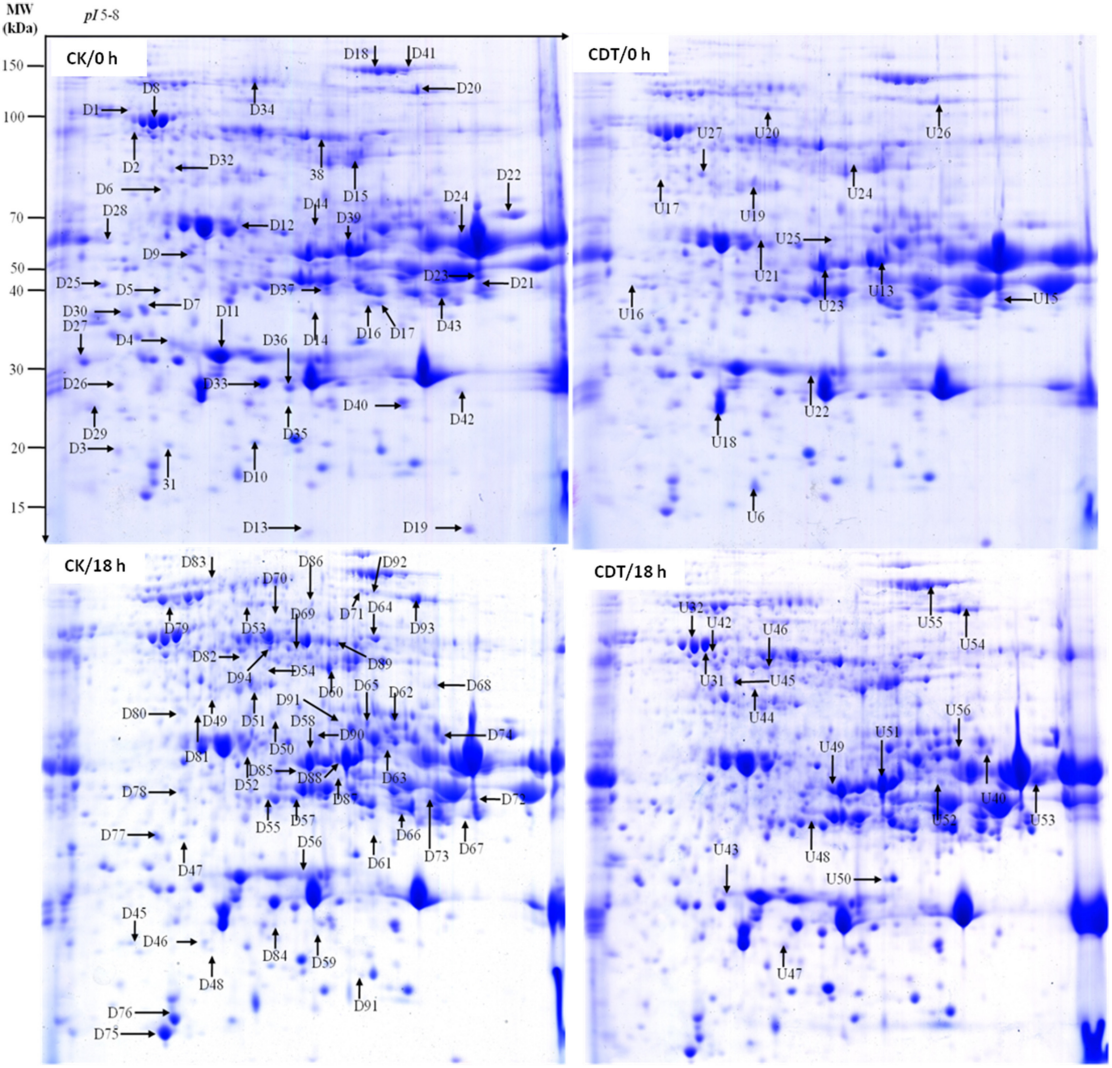
**Representative two-dimensional gel images of *Brassica napus* seeds**. Gels are from both control (left panel) and treated (right panel) seeds at 0 and 18 h after imbibition. Arrows show the differentially displayed protein spots, D and U stands for down-regulated and up-regulated proteins, respectively.

To further explore the molecular mechanisms underlying the loss of seed vigor due to artificial aging treatments; comparative proteomic analysis was carried out. Based on the germination assay result, CK and CDT seeds showed obvious difference in germination rate at 18 h of imbibitions (Figure 1), so proteins were isolated from the *B. napus* seeds at 0 h and 18 h after imbibition respectively. The proteins will then be resolved on 2-D PAGE and stained with CBB R-250. Three independent replicates were performed for each sample (Figures 4, S2). After staining, the gels were scanned and digitized for the comparison. Analysis of the 2-DE gels was conducted with PDQuest™ 2-DE Analysis software (version 8.0). Combined the biological replicates showed that there were about 600 reproducible protein spots (550–600) on each gel. A comparison of the 2-DE gels showed a total of 81 differentially accumulated spots (more then 2-fold changes in abundance) between CK and CDT samples, of which 36 and 48 were from 0 to 18 h of imbibition respectively. Three spots were common at both time points of imbibition.

**Figure 5:**
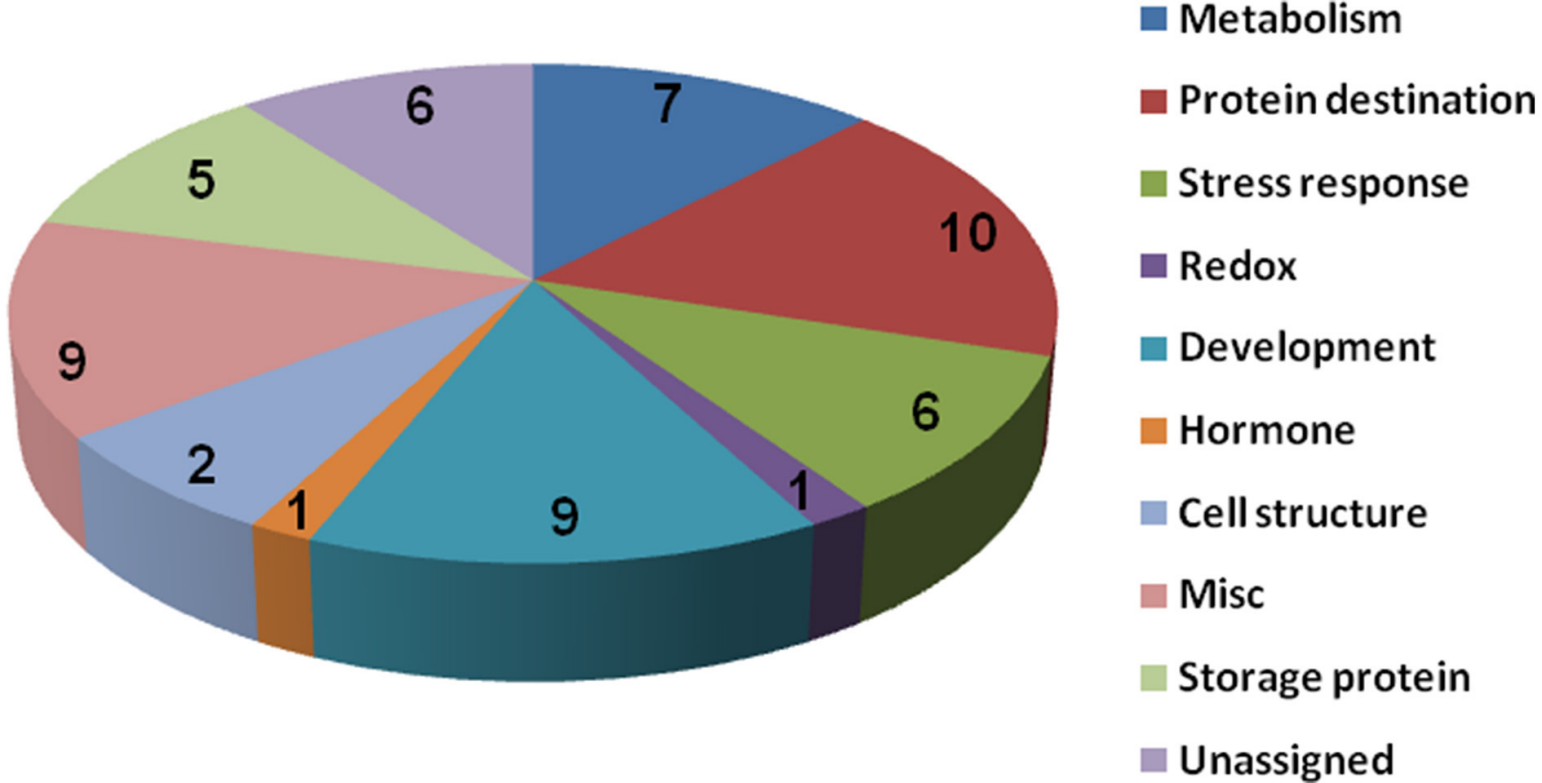
**Protein functional categories of the differentially displayed proteins**. Functional categorization was conducted according to MapMan (Thimm et al., 2004). The digital numbers stand for the number of proteins in each group.

To know if any of these differentially accumulated spots were involved in the seed aging process, these were excised from the gels, digested with trypsin and subjected to MALDI-TOF/TOF MS analysis. Based on the criteria described in materials and methods, 49 protein spots were successfully identified (Supplemental Table S1). Among these, seven spots were identified as two proteins, which resulted in the identification of a total of 54 distinct proteins (Table 1, Figure S3). Of these identified proteins, 15 proteins were only with the *B. napus* EST accession number. The sequences of these proteins were blasted against Arabidopsis database to gain a better understanding of their functions (Table 2). For many identified proteins, there were differences between their experimental and theoretical pI and molecular weight. Several possibilities may help to explain this result. First, the genome of *B. napus* is not fully sequenced and annotated, which might result in deviation; second, some of the proteins might be subjected to post-translational modifications or cleavage. MapMan ontology analysis (Thimm et al., 2004) sorted all the identified proteins into 10 functional groups including metabolism, protein destination, stress response, redox homeostasis, development, hormones related, cell structure, miscellaneous enzymes, storage proteins, and function unassigned proteins (Table 1, Figure 5).

**Table 1:** **Identification of the differentially displayed proteins in rapeseeds exposed to artificial aging treatment**.

**Protein ID**	**Accession**	**Description**	**Exp. *pI*/Mr**	**Theo. *pI*/Mr**	**Score**	SC^*^	Fold change^**^	
							**0 h**	**18 h**
**METABOLISM**								
D2^#^	gi|7525018	ATP synthase	5.54/79.79	5.19/55.35	129	6	0.4±0.12	1±0.12
U10	gi|131979	RuBisCO large subunit	6.64/51.92	6.23/51.98	276	9.5	0.97±0.05	4. 3±0.06
U19-1^#^	gi|3549670	Putative protein	6.04/69.15	6.34/47.12	49	2.8	9.3±6.2	1±0.06
D22	gi|166706	Glyceraldehyde-3-phosphate dehydrogenase	7.82/49.03	6.34/37.08	264	10.4	0.5±0.3	0.96±0.03
D38	gi|11587	Unnamed protein product	6.7/75.76	6.32/47.52	89	3.1	0.2±0.06	1±0.18
D44	gi|2497857	Malate dehydrogenase Precursor	6.5/42.72	8.81/35.86	187	8	0.22±0.03	0.2±0.03
D82-1	gi|13605559	AT3g03250/T17B22_6	5.82/69.32	5.93/51.85	81	5	0.92±0.03	0.42±0.07
**PROTEIN DESTINATION**								
D13	gi|3086	UBI 3 fusion protein	6.51/10.41	9.82/17.34	80	8	0.15±0.04	0.92±0.06
D26-1	gi|297796499	Ubiquitin1	5.34/21.31	5.14/17.83	72	7	0.37±0.15	1.1±0.21
U45-1^#^	gi|224119900	Predicted protein	6.02/69.12	5.8/48.35	98	4	1±0.1	2±0.95
U45-2^#^	gi|50814	Unnamed protein	6.02/69.12	5.4/44.69	87	4	1±0.1	2±0.95
D69	gi|224119900	Predicted protein	6.44/62.75	5.8/48.35	81	4	0.5±0.21	0.4±0.018
D70	gi|14423532	Putative chaperonin	6.33/73.01	5.83/59.46	83	3	1±0.05	–∞
D81	gi|62321134	Hypothetical protein	5.75/51.4	5.55/40.59	208	7	1±0.17	0.49±0.02
D82-2	gi|1709798	26S protease regulatory subunit 6B homolog	5.82/69.32	5.3/46.67	62	2	0.92±0.03	0.42±0.07
D87-1	gi|134037046	Threonine endopeptidase	6.65/34.97	5.79/28.25	81	7	1.1±0.16	0.5±0.04
D87-2	gi|2511592	Proteasome component	6.65/34.97	5.77/27.55	65	7	1.1±0.16	0.5±0.04
**STRESS RESPONSE**								
D27	gi|2326354	LMW heat shock protein	5.12/24.35	7.88/23.59	81	4.3	0.2±0.09	1.1±0.18
D33	gi|16340	Unnamed protein product	6.27/21.82	5.21/17.44	47	5.8	0.5±0.02	0.98±0.13
D36	gi|157849708	17.6 kDa class II heat shock protein	6.44/21.28	7.8/17.60	67	5.8	0.46±0.02	1.1±0.03
U42	AM058901	AM058901.1 Brassica rapa	5.83/23.52	5.47/15.28	84	12	1.0±0.17	3.7±1.57
D79	gi|115448989	Os02g0774300	5.59/88.24	5.49/73.08	192	4	1.0±0.11	0.3±0.07
D83	gi|1906826	Heat shock protein	5.8/98.62	4.97/80.23	96	3	0.99±0.09	0.43±0.08
**REDOX**								
D26-2	gi|4928472	Type2 peroxiredoxin	5.34/21.31	5.37/17.55	53	6	0.4±0.15	1.1±0.21
**DEVELOPMENT**								
D15	CX267648	CX267648.1 Brassica rapa	6.82/63.74	7.19/22.34	71	9	1.0±0.18	–∞
U18-1	DY004591	DY004591.1 Brassica rapa	5.87/19.93	9.12/26.25	239	12	2.9±0.86	–∞
D30-1	DY003072	DY003072.1 Brassica rapa	5.39/31.11	5.04/22.93	78	9	0.4±0.04	0.98±0.07
D30-2	CD828227	CD828227.1 Brassica rapa	5.39/31.11	7.94/13.25	68	17	0.4±0.04	0.98±0.07
D37^#^	gi|15226403	Cupin family protein	6.63/29.66	7.1/55.90	79	4.8	0.27±0.16	1.1±0.07
U44	EV179242	EV179242.1 Brassica rapa	5.81/63.46	9.71/31.49	69	5	1.0±0.2	3.3±0.79
D74^#^	DY001490	DY001490.1 Brassica rapa	7.39/42.53	9.78/22.11	72	9	1.0±0.08	0.3±0.12
D76^#^	gi|461840	Cruciferin CRU1	5.57/11.55	7.64/56.87	287	5	1.0±0.04	0.5±0.07
D90	DY002799	DY002799.1 Brassica rapa	6.68/43.73	5.16/22.40	175	20	1.0±0.09	0.2±0.12
**HORMONE RELATED**								
D31	gi|556805	Em protein	5.66/15.8	6.75/9.94	245	28.3	0.26±0.02	1.0±0.12
**CELL STRUCTURE**								
U27^#^	gi|48527433	Actin	5.77/65.64	5.24/41.95	156	10	2.9±0.91	1.0±0.11
U43^#^	gi|6628	Actin	5.87/60.76	5.3/42.02	102	4	1.0±0.09	3.1±0.72
**MISCELLANEOUS ENZYMES**								
D11^#^ D39	gi|12322163	Dormancy related protein	6.57/41.16	5.92/31.41	88	3.8	0.54±0.11	1.0±0.16
U13^#^ D64	gi|34222076	Mannose-binding lectin superfamily protein	6.9/43.39	6.03/49.25	87	4	4.1±1.13	1.1±0.07
U19-2^#^	gi|159470791	Glycosyltransferase	6.04/69.15	8.95/57.22	46	2.8	9.6±6.2	1.0±0.06
U26-2	gi|757740	Beta-glucosidase	7.19/81.48	6.21/58.47	112	2.5	2.5±0.56	1.0±0.10
U50^#^	gi|12322163	Dormancy related protein	6.71/37.1	5.92/31.41	95	3	1.0±0.11	2.1±0.62
D88^#^	gi|12322163	Dormancy related protein	6.65/38.64	5.92/31.41	74	3	1.0±0.17	0.62±0.27
D92	gi|757740	Beta-glucosidase	6.89/92.86	6.21/58.92	72	3	1.0±0.08	274±26
**STORAGE PROTEINS**								
D37	gi|15226403	Cupin family protein	6.63/29.66	7.1/55.90	79	4.8	0.5±0.16	1.0±0.11
U41	gi|167136	Cruciferin precursor	5.68/72.89	6.84/56.43	92	3	0.99±0.08	2.5±0.01
D75	gi|166678	12S storage protein CRB	5.5/10.82	6.77/50.95	158	3	0.98±0.11	0.56±0.07
D84	gi|166678	12S storage protein CRB	6.19/17.83	6.77/50.95	79	3	1.0±0.10	172±34
D85^#^	gi|166678	12S storage protein CRB	6.4/37.1	6.77/50.95	63	3	1.0±0.05	0.33±0.13
**UNASSIGNED**								
D7	DY002554	DY002554.1 Brassica rapa	5.46/31.37	8.71/16.29	231	22	0.46±0.03	0.43±0.07
U18-2	DY002789	DY002789.1 Brassica rapa	5.87/19.93	9.35/24.44	205	14	2.9±0.39	–∞
D41	DY002558	DY002558.1 Brassica rapa	7.2/110.01	5.29/24.81	78	8	0.4±0.20	1.0±0.08
U48^#^	CX278338	CX278338.1 Brassica rapa	6.4/38.37	9.03/27.20	276	18	0.99±0.15	2.5±0.64
D71	DY002554	DY002554.1 Brassica rapa	6.88/84.95	8.71/16.29	100	13	1.1±0.21	–∞
D78	DY002558	DY002558.1 Brassica rapa	5.64/32.73	5.29/24.81	83	8	0.97±0.07	–∞

* *SC stands for sequence coverage*.

** *Fold changes are Means±SE from three replicates; –∞ stands for the disappeared spots in the related samples. D and U in protein ID list stand for down-regulated protein and up-regulated protein respectively*.

# *Indicates these proteins were statistically not but manually checked to be differentially expressed*.

**Table 2:** **Blast of the identified proteins through searching against *Brassica napus* EST database**.

**Protein ID**	**Accession no**.	**AGI No**.	**Description**	**Score**	**Identity (%)**	**Functional group**
D7	DY002554	AT2G42560	LEA domain-containing protein	79	47	Unassigned
D15	CX267648	AT2G36640	Embryonic cell protein 63	164	51	Development
D30-1	DY003072	AT2G28490	RmlC-like cupins superfamily protein	266	76	Development
D30-2	CD828227	AT2G28490	RmlC-like cupins superfamily protein	79	63	Development
D41	DY002558	AT2G42560	LEA domain-containing protein	172	55	Unassigned
D58	DY001351	AT5G45690	Protein of unknown function (DUF1264)	369	82	Unassigned
D74	DY001490	AT3G22640	Cupin family protein	185	68	Development
D71	DY002554	AT2G42560	LEA domain-containing protein	78.6	47	Unassigned
D78	DY002558	AT2G42560	LEA domain-containing protein	172	55	Unassigned
D90	DY002799	AT3G22640	Cupin family protein	290	70	Development
U18-1	DY004591	AT1G03890	RmlC-like cupins superfamily protein	299	78	Development
U18-2	DY002789	AT5G01300	PEBP (phosphatidylethanolamine- binding protein) family protein	302	85	Unassigned
U42	AM058901	AT2G21060	Glycine-rich protein 2B	145	84	Stress
U44	EV179242	AT3G53040	LEA domain-containing protein	117	52	Development
U48	CX278338	AT5G45690	Protein of unknown function (DUF1264)	277	82	Unassigned

### Effects of ABA and GA on the seed aging

**Figure 6:**
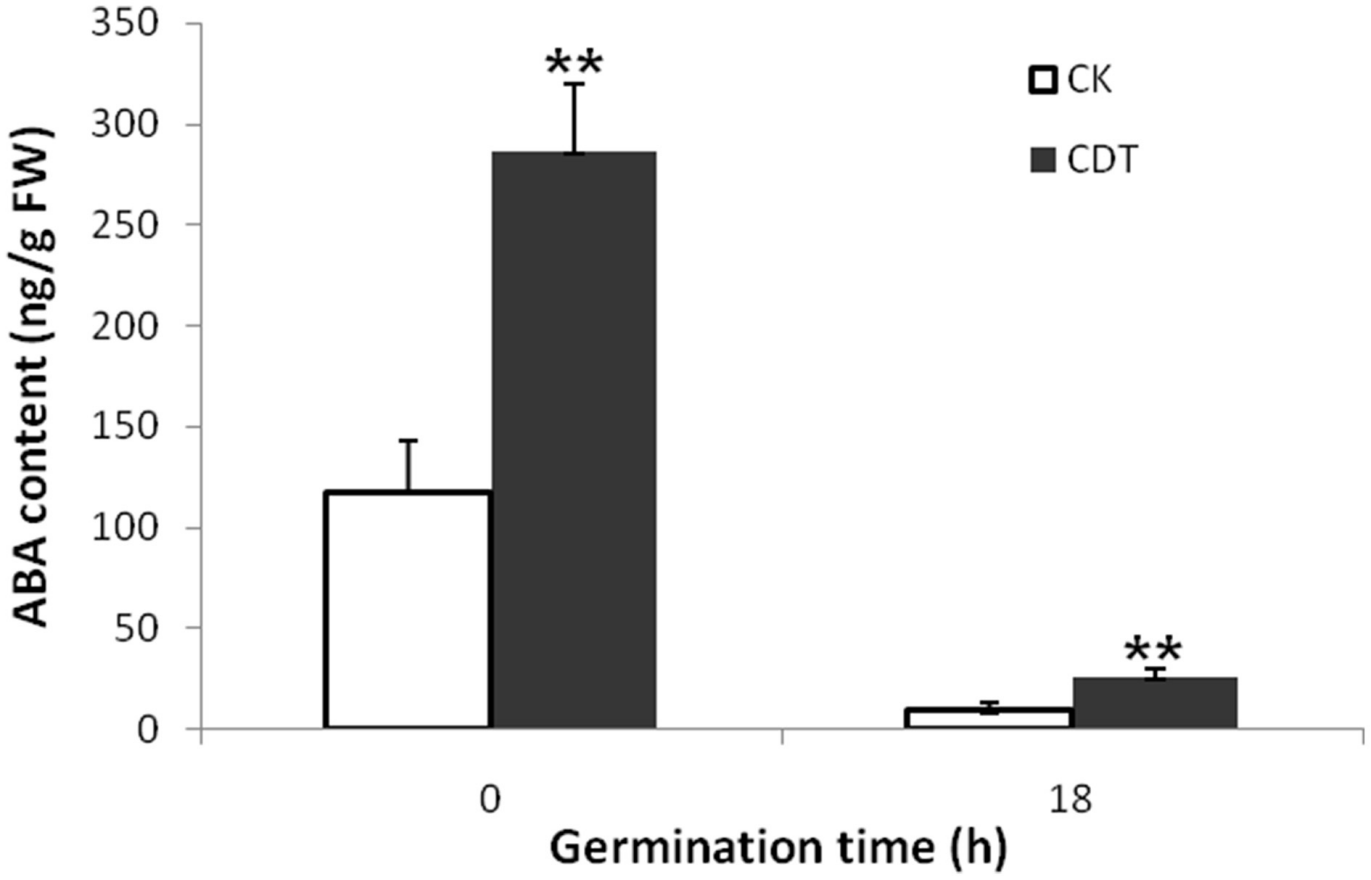
**Contents of ABA in *Brassica napus* seeds exposed to CDT treatment during germination**. Values are the means ± SE from three biological replicates. ^**^ indicate significant difference at *P* < 0.01.

**Figure 7:**
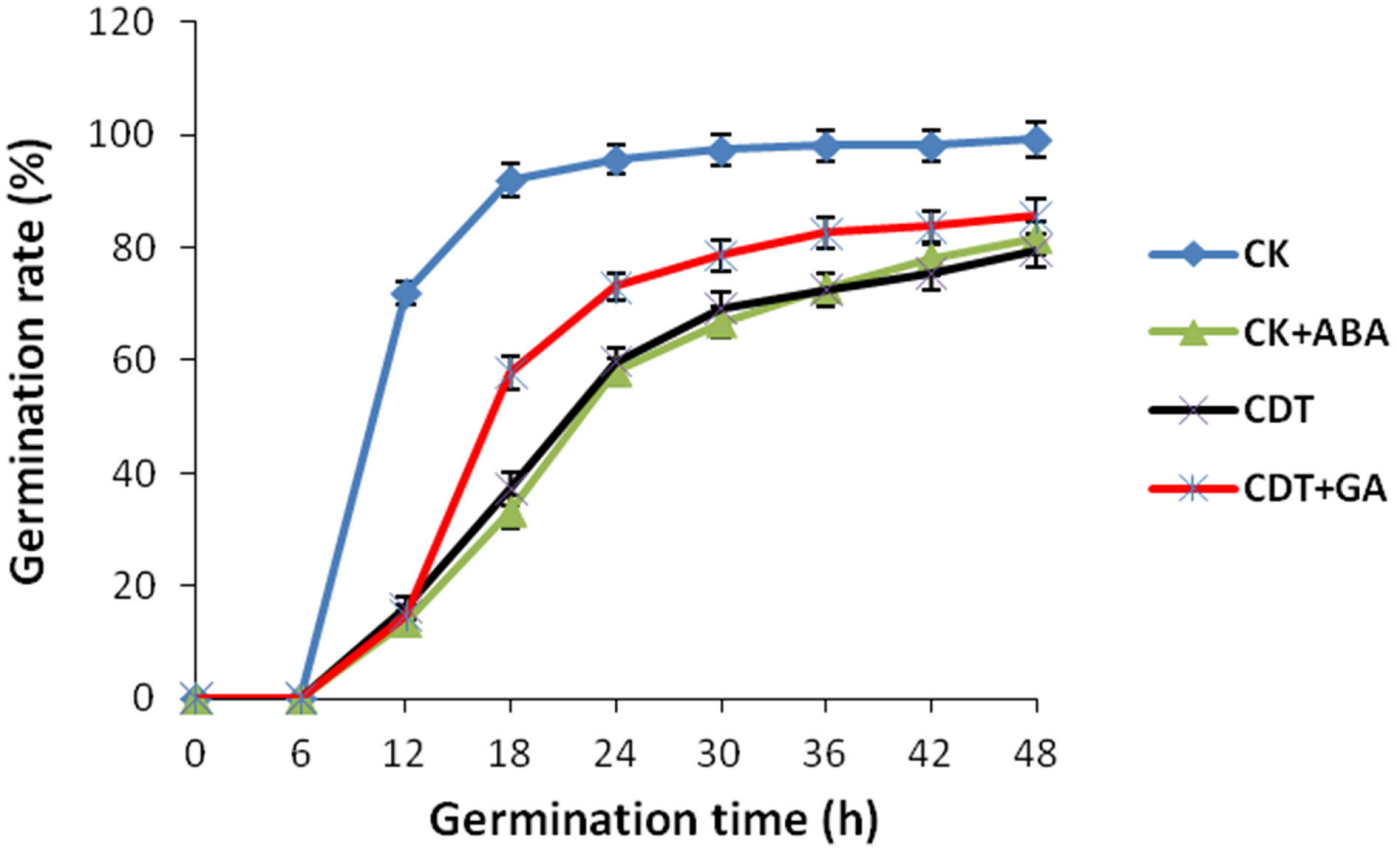
**Effects of ABA and GA on *Brassica napus* seed germination**. Values are the means ± SE from three biological replicates.

As no major differences in the ROS concentrations and antioxidant enzymes activities were observed after CDT treatment, we speculated that oxidative stress is not the major factor involved in the inhibition of germination after CDT treatment. Therefore, involvement of some other factors, which resulted in delayed germination after CDT treatment, was expected. Among all the external and internal factors, ABA seems to be the most important one which inhibits seed germination. Therefore, we measured the ABA content of the CK and CDT seeds at both 0 and 18 h after imbibition. Interestingly, the ABA content of the CDT seeds was much higher than that observed in the CK seeds at both the time points, and showed a sharp decrease upon imbibition in both the samples (Figure 6), suggesting involvement of ABA in seed aging. To further confirm this hypothesis, the CK seeds were germinated in the presence of ABA, and showed a delayed germination (Figure 7). It is known that GA and ABA play antagonistic roles in regulating seed germination, so we also germinated the CDT seed in the presence of GA to see if this treatment can recover its germination phenotype or not. Consistently, GA treated CDT seeds showed partially recovered germination (Figure 7).

## Discussion

Temperature and moisture content (relative humidity) are two important environmental factors that influence seed aging. High temperature and moisture accelerate the process of seed aging, leading to the rapid loss of seed vigor. The accelerated aging has been applied as an indicator of crop seed storability (Priestley, 1986). To study the mechanism of seed aging, researchers have developed the accelerated aging protocol in laboratory by exposing the seeds to high temperature and humidity conditions (Job, 2005; El-Maarouf-Bouteau et al., 2011). ROS have been presumed as the main factors which lead to the seed aging during storage (Bailly et al., 1996; Bailly, 2004; Kibinza et al., 2006; Parkhey et al., 2012). In order to understand the mechanisms underlying *B. napus* seed aging, especially at the initiation stage, we subjected the *B. napus* seeds with CDT, which obviously delayed the germination and slightly decreased the germination rate which was similar to the 1 year naturally aged seeds (Figure 1). These results suggested that this treatment was suitable for further analysis as it could mimic the physiology of 1 year naturally aged seeds.

The CDT resulted in the increased ion leakage (Figure 2), which reflects the damages of cellular membranes. This damage, in turn, resulted in the accumulation of MDA in the CDT seeds (Figure 2). Previously, it was considered that attack of ROS on membranes might be one of the reasons that lead to the ion leakage (Leymarie et al., 2012). Furthermore, previous study also showed that high temperature and humidity drastically increased the extent of protein oxidation in Arabidopsis seeds (Rajjou et al., 2008). However, after the CDT in the *B. napus* seeds, we did not detect any over accumulation of ROS (Figure 3A), and any differentially accumulated antioxidant enzymes except for peroxiredoxin (Table 1). Since only about 500 proteins were detected, it could not be absolutely excluded that there were some antioxidant enzymes changed. In spite of this, CDT did decrease the rate of ROS detoxification during germination (Figure 3A), suggesting that artificial aging might disturb the ROS scavenging system. This result was also supported by the decline of SOD and CAT activities (Figure 3B). The CDT might decrease the activities of the antioxidant enzymes because of the high temperature, since most of the enzymes have their highest activities at around 35°C under physiological conditions.

Functional classification of the identified proteins showed that proteins related to protein modification and destination, development and cell structure were differentially modulated after CDT. However, previous studies on pea (Barba-Espin et al., 2011) and wheat seeds (Bykova et al., 2011) have shown that H_2_O_2_ treatment preferentially regulates proteins majorly involved in redox homeostasis, metabolism, hormones related and RNA related, and it could invoke the changes of proteins with similar functions in different tissues (Wan and Liu, 2008; Zhou et al., 2011). The differences in the differentially accumulated proteins in this study with the previously published reports on pea and wheat, suggest that the seed aging mechanism in the *B. napus* seeds is quite different with that indicating in case of pea and wheat seeds. Our results suggest that CDT treatment might initiate the aging of *B. napus* seeds through enhancing the biosynthesis of germination inhibitors and lately through the accumulation of ROS. Although there were 81 differentially accumulated proteins between CK and CDT seeds, only 49 spots were successfully identified. The identification rate is just about 60%, which might be explained by the un-sequenced genome of *B. napus*. Moreover, there seven spots were identified as two different proteins, which should result in difficulties to evaluate a real abundance of each protein within them. This is one of the limitations in 2-D gel based proteomic techniques. Future studies with gel-free systems might help to provide more information.

Since the CDT treatment delayed the germination not primarily because of accumulation of ROS, we speculated that there should be some other mechanisms which mediate the seed aging process in *B. napus* seeds. Among the identified proteins, most of the proteins involved in metabolism and protein destination were decreased in the CDT seeds. These results indicate that the basic biological activities might be inhibited by the CDT treatment, which would result in the delayed germination. Interestingly, rubisco large subunit was increased in the CDT treated seed at 18 h after germination, which indicates the existence of feedback regulation. In contrast to the metabolism and protein destination related proteins, many cell structural proteins and miscellaneous enzymes, such as actin, mannose-binding lectin superfamily protein, glycosyltransferase, beta-glucosidase, were increased. All these proteins are related to the cell and cell wall structures. It might be interesting to know how these proteins affect seed aging.

Among all the internal factors, ABA has been reported to be one of the main factor that inhibits the seed germination (Gubler et al., 2005; Finch-Savage and Leubner-Metzger, 2006; Penfield et al., 2006). Measurements of the ABA content in *B. napus* seeds showed that there was a sharp increase of ABA content in the CDT treated seeds (Figure 6). Although the ABA was degraded during the germination, its concentration was still higher in the CDT treated seeds in comparison with the CK seeds (Figure 6). Furthermore, imbibition of the CK seeds with ABA solution showed delayed germination (Figure 7). Germination of the CDT treated seeds was partially recovered after the GA treatment, further confirming the involvement of ABA in the inhibition of seed germination during aging. However, how this ABA concentration is increased during aging treatment in the *B. napus* seeds, is still elusive. Unfortunately, we did not detect any changes of the enzymes involved in the ABA biosynthesis and degradation. It is well known that ABA is synthesized in the seeds during desiccation which allow the survival of seeds in dry state (Tan et al., 1997; Nakabayashi et al., 2005). During this process, genes involved in ABA biosynthesis were found to be highly expressed, and corresponding enzymes were abundantly accumulated. Based on these results, we suggest that exposure of seeds with high humidity and temperature might partly recover the activities of the ABA biosynthesis enzymes which results in the enhanced production of ABA in the *B. napus* seeds during aging.

## Author contributions

YX did the experiments; HD prepared the seeds and analyzed some of the data; YP designed the experiments and wrote the manuscript.

### Conflict of interest statement

The authors declare that the research was conducted in the absence of any commercial or financial relationships that could be construed as a potential conflict of interest.

## Supplementary material

Table S1 **Identification of the differentially expressed proteins**.
