# Supplementary figures and images for: Physiological and proteomic analyses on artificially aged Brassica napus seed
Source: Front Plant Sci. 2015 Feb 25;6:112. doi: 10.3389/fpls.2015.00112 (PMC4340179; doi:10.3389/fpls.2015.00112)

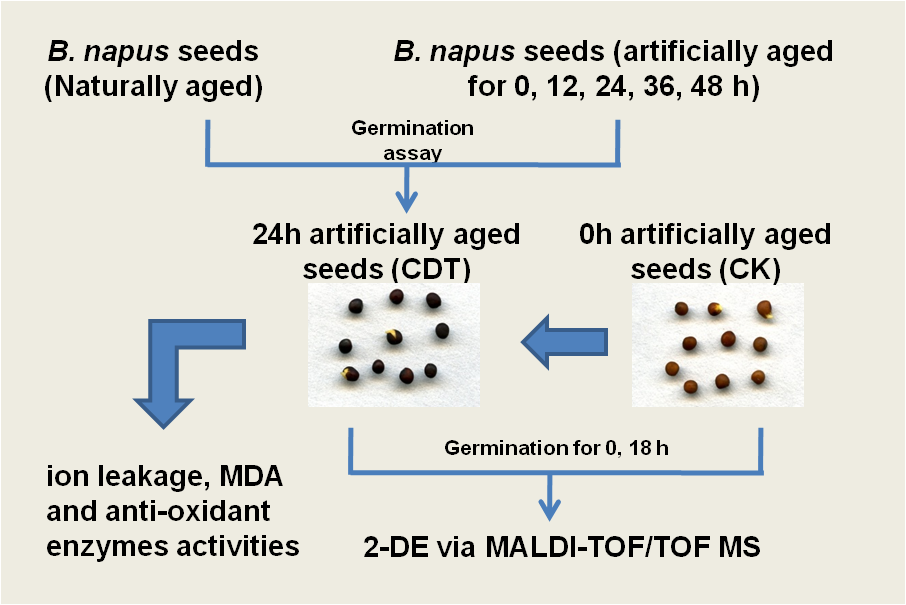

Supplement: Supplementary file 2 [file Image1.TIF]
