# Supplementary material for: Physiological and proteomic analyses on artificially aged Brassica napus seed
Source: Front Plant Sci. 2015 Feb 25;6:112. doi: 10.3389/fpls.2015.00112 (PMC4340179; doi:10.3389/fpls.2015.00112)
Supplement: Supplementary file 3 [file Image2.PDF]

Figure S2 2-D gel images of triplicates experiments. CK stands for the control, CDT stands for the 24 h of ageing treatment.

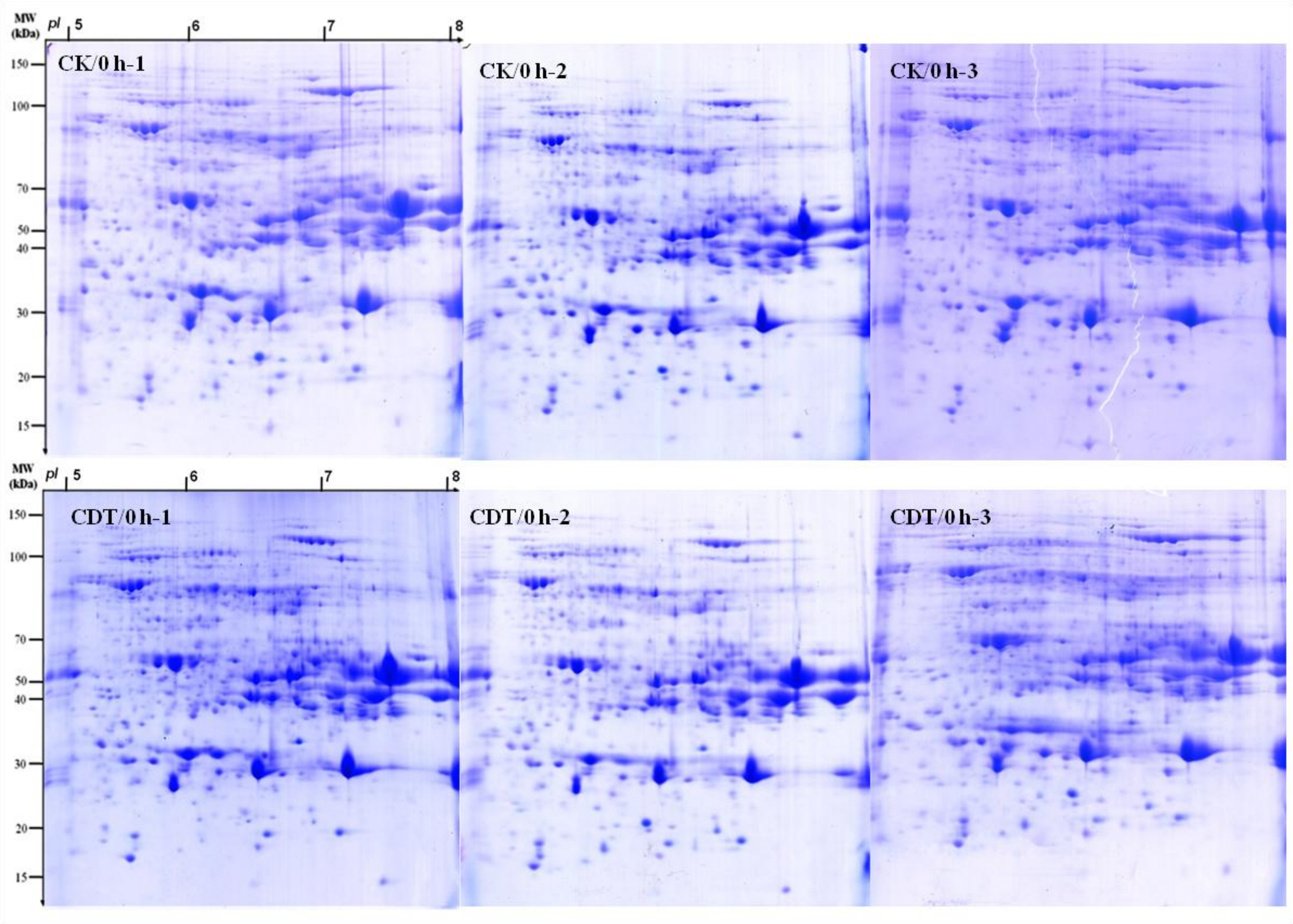

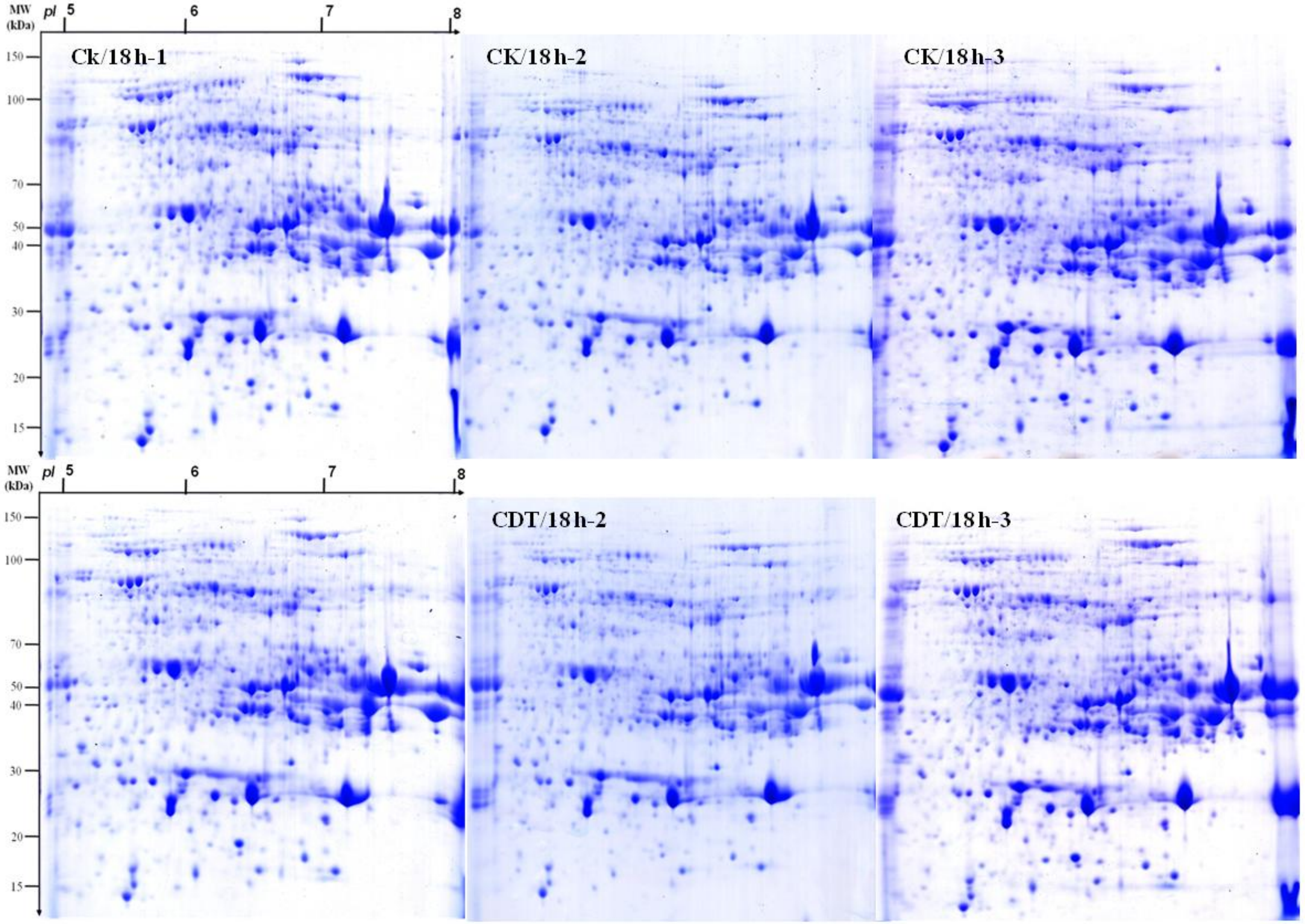
MW (kDa)
pI
5
6
7
8
150
100
70
50
40
30
20
15
Ck/18h-1
CK/18h-2
CK/18h-3
CDT/18h-2
CDT/18h-3
